# Spectrally Tunable Neural Network-Assisted Segmentation of Microneurosurgical Anatomy

**DOI:** 10.3389/fnins.2020.00640

**Published:** 2020-06-30

**Authors:** Sami Puustinen, Soukaina Alaoui, Piotr Bartczak, Roman Bednarik, Timo Koivisto, Aarno Dietz, Mikael von und zu Fraunberg, Matti Iso-Mustajärvi, Antti-Pekka Elomaa

**Affiliations:** ^1^School of Medicine, Faculty of Health Sciences, University of Eastern Finland, Kuopio, Finland; ^2^School of Computing, Faculty of Science and Forestry, University of Eastern Finland, Joensuu, Finland; ^3^Department of Neurosurgery, Neurocenter, Kuopio University Hospital, Kuopio, Finland; ^4^Department of Otolaryngology, Kuopio University Hospital, Kuopio, Finland; ^5^Eastern Finland Center of Microsurgery, Kuopio University Hospital, Kuopio, Finland

**Keywords:** microsurgery, neurosurgery, narrow-band imaging, machine learning, optimal bands, spectral imaging analysis, anatomy, endoscopy

## Abstract

**Background:**

Distinct tissue types are differentiated based on the surgeon’s knowledge and subjective visible information, typically assisted with white-light intraoperative imaging systems. Narrow-band imaging (NBI) assists in tissue identification and enables automated classifiers, but many anatomical details moderate computational predictions and cause bias. In particular, tissues’ light-source-dependent optical characteristics, anatomical location, and potentially hazardous microstructural changes such as peeling have been overlooked in previous literature.

**Methods:**

Narrow-band images of five (*n* = 5) facial nerves (FNs) and internal carotid arteries (ICAs) were captured from freshly frozen temporal bones. The FNs were split into intracranial and intratemporal samples, and ICAs’ adventitia was peeled from the distal end. Three-dimensional (3D) spectral data were captured by a custom-built liquid crystal tunable filter (LCTF) spectral imaging (SI) system. We investigated the normal variance between the samples and utilized descriptive and machine learning analysis on the image stack hypercubes.

**Results:**

Reflectance between intact and peeled arteries in lower-wavelength domains between 400 and 576 nm was significantly different (*p* < 0.05). Proximal FN could be differentiated from distal FN in a higher range, 490–720 nm (*p* < 0.001). ICA with intact tunica differed from proximal FN nearly thorough the VIS range, 412–592 nm (*p* < 0.001) and 664–720 nm (*p* < 0.05) as did its distal counterpart, 422–720 nm (*p* < 0.001). The availed U-Net algorithm classified 90.93% of the pixels correctly in comparison to tissue margins delineated by a specialist.

**Conclusion:**

Selective NBI represents a promising method for assisting tissue identification and computational segmentation of surgical microanatomy. Further multidisciplinary research is required for its clinical applications and intraoperative integration.

## Introduction

Along with cutting-edge handcraft, visual perception is *sine qua non-for* surgical outcome. Precise delineation of pathological margins is imperative, especially in intracranial microsurgery, where errors may lead to severe neurological deficits. Unfortunately, several pathologies extend beyond the capabilities of the human visual system. To elucidate, the evaluation of blood flow and aneurysmal lesions are conventionally visualized with fluorescent colorants such as indocyanine green (ICG). Mere visual information is rudimentary also in oncological procedures as unquestionably complicated anatomy is conjoined with diffuse infiltrations into the healthy tissues. The tissues light interactions, such as reflection and scattering, render them in various shades of pink and red, thereby restraining the visual contrast available to the operating surgeon (Jacques, 1996).

Although fluorescence-guided surgery (FGS) has been the hallmark for neurosurgical optical imaging due to improvements in tumor resection rates (Hadjipanayis et al., 2015), experimental optical technologies such as diffuse reflectance spectroscopy (DRS), Raman spectroscopy, and optical coherence tomography have been implemented for neurosurgery in search of expanded indications. Both the FGS and DRS require contrast agents. FGS employs exogenous colorants, e.g., ICG, whereas in DRS, the contrast molecule is endogenous such as NADPH, which is involved with the metabolic pentose phosphate pathway. Similarly, Raman spectroscopy provides contrast endogenously by the inelastic scattering of photons interacting with tissue (Richards-Kortum and Sevick-Muraca, 1996). Optical coherence tomography uses back-reflected or backscattered light to reconstruct tomographic images of tissues in millimeter depths (Fujimoto et al., 2000). In clinical neurosurgery, these methods have seemed most applicable for functional measurements or delineation of tumors (Lin et al., 2001; Böhringer et al., 2009; Lu et al., 2016). The intraoperative implementation of these techniques requires complex visualization techniques and contact handheld probes that may compromise dexterity.

As a novel solution, narrow-band imaging (NBI) and spectral imaging (SI) systems can enhance the contrast and aid segmentation of different regions of interest (Panasyuk et al., 2007). These techniques are more universally applicable and can also enhance the other imaging systems, such as ICG-FGS in the analysis of cerebral perfusion (Li et al., 2019). SI methods are emerging in medicine, and novel applications include early detection of tumor margins and mucosal changes and retinal disease and the assessment of tissue perfusion (Mordant et al., 2011; Gerstner et al., 2012; Zheludev et al., 2015; Holmer et al., 2018). Along with their possible diagnostical advantages, SI systems have useful properties for intraoperative use. In contrast to most optical technologies, these systems are non-ionizing, are non-invasive, and do not require an extrinsic colorant to visualize important structures. The design of SI systems is often flexible, and the captured data can be modified both computationally and optically to highlight the regions of interest (Levenson et al., 2012). The primary disadvantages of SI systems are speed, high expense, and complexity, which arise from the fact that the technique demands high-performance computers, quick-to-react detectors, and storage for a significant amount of multidimensional data (Landgrebe, 2002). Equipment and the tissues themselves seem to cause significant variability in spectral responses, and the observed differences are often minor, e.g., close curves instead of sharp peaks, so careful calibration of the SI systems is required (Li et al., 2013). Therefore, data sciences are closely connected to SI, and machine learning paradigms and data mining are important tools for research (Han et al., 2011). Adding to uses in medical image analysis, artificial neural networks are particularly viable for optic tissue margin delineation in surgeries (Dong et al., 2017; Halicek et al., 2017).

A typical SI system is composed of a light source, optic lenses, dispersive elements such as grating or a prism, and a detector (Boldrini et al., 2012). These systems collect reflected light in a way that every imaging pixel includes a selected spectrum for that location. The acquired data cube results in three dimensions (*x*, *y*, and λ), and in this way, both quantitative and locational data are presented. The amount of information is exponential compared to conventional cameras, and computational models are used to manipulate the data in order to find feasible targets for applications such as NBI. The light interactions depend heavily on the molecular constituents, and consequently, the collected spectral information forms a characteristic spectral signature that is suitable for objective identification. Various biological and pathological processes modify the cellular and molecular states of the tissue, and thereby, a change in the spectra is expected (Tuchin and Tuchin, 2007; Piña-Oviedo et al., 2017). Overall, spectral analysis has been around for years, and it has many successful applications in different fields of science such as forensics, food quality analysis, and several industrial sectors (Thumm et al., 2010; Edelman et al., 2012; Huang et al., 2014).

At present, preclinical-phase SI studies focus on histological sections, animal models, or tissue phantoms. Adding to oncologic resections, such studies have documented segmentation of normal surgical anatomy (Akbari, 2009; Nouri et al., 2016). Among surgical pathology identification, Akbari et al. (2010) demonstrated the identification of intestinal ischemia on a porcine model in surgical conditions. Neurosurgical use of SI systems has been poorly documented, as intraoperative integration in general. Few promising trials suggest that when properly translated to the operation room, SI systems could assist the delineation of brain tumors and increase the removal efficacy (Piñeiro et al., 2017; Fabelo et al., 2018a, b). SI methods have also been used to assess cerebral cortex oxygenation and the hemodynamic responses (Pichette et al., 2016; Giannoni et al., 2018). However, the number of publications addressing microsurgical anatomical tissues’ optic characteristics is low. It has been omitted how cranial location moderates the optical properties and whether these features vary individually. Bartczak et al. proposed and tested a portable system for on-site medical SI (Hadjipanayis et al., 2015). In this study, the spectral properties of *ex vivo* bone, dura mater, muscle, fat, and carotid artery all exhibit a unique spectral signature. The authors considered intraoperative requirements and highlighted that short capture times are required for operation room conditions (Bartczak et al., 2018). In particular, liquid crystal tunable filter (LCTF)-based systems appear clinically feasible since they do not require mechanical filter adjustment and enable band selection (Gebhart et al., 2005; Nouri et al., 2014).

Spectrally tunable light sources can provide better illumination for the tissue’s visual analysis. Tuning may help to optimize computational time, may simplify SI systems, and, ultimately, may lead to better cost efficacy with less errors. This technology may also be feasible for identification of tissue layers or minuscule alterations in tissues, such as trauma or plaque formation in arteries. Similarly, meningeal folding and central nervous system myelinization present potential examples of surgical anatomy identification. Of special interest are tissues that vary by structure and location, such as cranial nerves and arteries.

In our study, we determine the most significant wavelength ranges to identify internal carotid artery (ICA) and facial nerve (FN) samples subtle features based on anatomical location and iatrogenic damage. It is of interest to characterize whether these subtle features associated to optically defined changes in terms of variance and visible range reflectance. The results from this study could be used to modify spectral sensors according to optimal bands and reliable calibration, which are key factors for adopting new SI systems to neurosurgery. Optimized identification of neurovascular pathologies, such arterial wall remodeling in unruptured aneurysms or due to manipulation, could improve surgical outcomes and ease the recovery of patients, leading to increased patient satisfaction.

Our current work aims the following:

I To assess the spectral images’ discriminatory power and determine the most significant wavelength ranges for identification between ICA and FN samples.
II Report the adequacy of VIS (visible light) SI-based machine learning paradigm for tissue classification.

## Materials and Methods

### Tissue Preparation

This study was carried out in accordance with the recommendations of the Ethics Committee, Hospital District of Northern Savo. The protocol was approved by the Hospital District of Northern Savo. The study had approval for use of cadaveric tissue (Valvira decision no. 9202/06.01.03.01/2013) and fulfilled the Helsinki Declaration for ethical use of human material.

We studied the optic spectra of five (*n* = 5) *ex vivo* ICA and FN tissue samples extracted from freshly frozen cadaveric temporal bones, which were selected from the Kuopio University Hospital Department of Clinical Pathology supply. Both tissues carry a complex segmental anatomy and are key elements in microsurgery of the temporal bone (Gibo et al., 1981; Salame et al., 2002). After defrosting the samples, an ENT specialist, Iso-Mustajärvi (Fujimoto et al., 2000; Lin et al., 2001) extracted the distal and proximal part of the FN and cervical and petrous part of the ICA from the chosen samples. The extraction was performed by using common microsurgical instruments and high-speed drill under an operating microscope. Following the sample extraction, tissue-specific anomalies were documented.

The ICAs were cut skew, and the outermost layer, tunica adventitia, was removed carefully distally but left intact on the far side. The FN was collected from intracranial and intratemporal parts of the temporal bone in order to investigate differences especially caused by the epineurium, the outermost layer of connective tissue surrounding peripheral nerves, which protects the nerve and is presumed to increase after it enters the facial canal (Salame et al., 2002).

Both the peeled and normal samples of ICA and proximal and distal parts of FN were attached to a dark cardboard to control background reflectance. Each cardboard included ICA peeled, ICA normal, FN proximal, and FN distal samples, and the set was identified by a color (black, blue, red, white, and yellow, respectively). After fixing, the samples were covered with gauze swabs moistened with isotonic saline to prevent drying.

The fixed tissue samples were annotated according to the prepared margins by using the VGG Image Annotator 2.0.5. software (Dutta and Zisserman, 2019).

### System Description and Design

**FIGURE 1:**
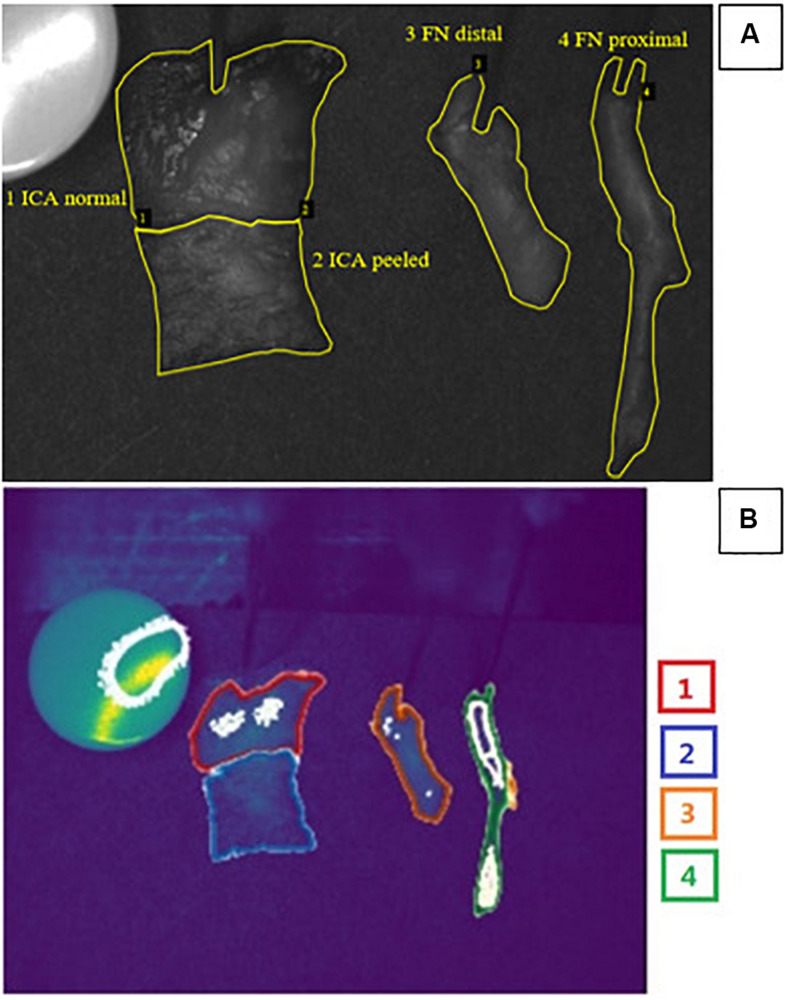
**(A)** Example of a grayscale image for spectral bands at 555 nm used for annotations of measured *ex vivo* tissues made by an ENT specialist. Manually classified area from tissues (Jacques, 1996; Richards-Kortum and Sevick-Muraca, 1996; Fujimoto et al., 2000; Hadjipanayis et al., 2015) was used as a ground truth. **(B)** Automatic segmentation results for corresponding example obtained via the combination of U-Net convolutional neural network and preselected optimal spectral bands. Color of the borders corresponds to tissue types: red, ICA normal; blue, ICA peeled; orange, FN distal; and green, FN proximal. Of the pixels 90.93% were classified correctly. Grainy white parts of the image represent noise. Abbreviations: ICA, internal carotid artery; FN, facial nerve.

The reflectance data were gathered and analyzed within annotated margins presented in Figure 1A. The utilized system consisted of a stage for the fixed specimen, a monochrome CMOS camera (Thorlabs 3240CP-M, Thorlabs, Inc.) providing high-resolution images (1,280 × 1,024 pixels), a broadband halogen lamp and fiber (Thorlabs OSL2, Thorlabs, Inc.), and an LCTF (VariSpec VIS-20, CRi, Inc.) tuning the light source. The tuning range of the filter was 400–720 nm.

The single-output fiber bundle (Thorlabs OSL2FB, Thorlabs, Inc.) was used to guide the light to the entrance port of the LCTF device. The light from the fiber was collimated by using the collimating package (Thorlabs OSL2COL, Thorlabs, Inc.), filtered, and focused into a microscope ring illuminator (Thorlabs FRI61F50, Thorlabs, Inc.). The ring illuminator provides a uniform, 360°, shadow-free illumination area. The c-mount lens (Tamron Co., Ltd) and manual linear translation stage were used to zoom and position the camera. The synchronization of the LCTF device and the camera was implemented in custom C++ scripts and allowed for an automatic image sequence acquisition.

The setup was completely enclosed into a mobile electronics cart (Knürr EliMobil). Light-engaging material was used inside for suppression of stray light beams. In addition, a light-absorbing cloth was used to cover the cart to prevent the surrounding light pollution from distorting the measurement. The experimental setup, including image calibration and schematics, has been thoroughly described by Bartczak et al. (2018).

The complete imaging consisted of successive grayscale (monochrome) frames obtained while tuning a peak wavelength of the LCTF across its spectral range (400–720 nm) with increments of 2 nm. The exposure times were adjusted to compensate the differences in the system sensitivity. Identical exposure times were used to acquire white and dark references, needed for spectral reflectance as described before. Complete scanning across all filters resulted in 161 two-dimensional grayscale images that were employed to construct a three-dimensional (3D) spectral image cube. We collected four distinct tissue types from five different specimens, giving a total of 20 samples. Each sample was batched and analyzed from four different spots from over 161 wavelength bands, and this resulted in a total of 3,220 (4 × 5 × 4 × 161) bands to analyze. We chose the most optimal spectral bands for tissue identification and segmentation by using the statistical and computational methods described below.

### Statistical and Computational Methods

To achieve comparable illustrations of the optical behavior of different tissues, we normalized every spectrum by dividing the spectral data vector by its maximum values. Normality of the wavelength distributions was tested with the Shapiro–Wilk test. Mann–Whitney *U*-test was used to assess the differences of spectral reflectance between the batched images of the tissues because the assumption of normality was not fulfilled for every comparison. Statistical analyses were performed using SPSS 25.0 for Windows (IBM^®^ SPSS^®^ Statistics).

First, we attempted to identify optimal wavelengths for specific tissues. We utilized band reduction for identifying and discarding wavelengths that hinder image segmentation. The bands with the highest similarity coefficients were excluded to increase the visibility of spectral differences and to reduce the dimensionality of the data. Then both affinity and dissimilarity coefficients were considered for optimal band selection. The GDA-SS algorithm was used for the affinity measurements (Feng et al., 2017), and pairwise distance was applied to assess divergence (He and Niyogi, 2004). As this technique is sensitive to noise and outliers, we discarded one sample of ICA illustrating plaque formation. An image denoising technique, the Savitzky–Golay filter, was utilized to help the performance. Statistical tests report the similarity of distributions, while pairwise distances compared the spectra wavelength to wavelength, so it will highlight which bands indicate the difference better. Finally, this information was passed on to machine learning algorithms, as discussed below.

U-Net is convolutional neural network developed by Ronneberger et al. (2015) for the purpose of having a faster and more precise image segmentation. The convolutional architecture of U-Net was created with biomedical image segmentation problems in mind (Ronneberger et al., 2015). It consists of two paths. The first path is the contraction path (or the encoder). This layer is a traditional stack of convolutional and max pooling layers, and it is used to capture the context in the image. The second path is the symmetric expanding path (or the decoder). It only contains convolutional layers and no dense layers, which allows the path to accept images of any given size. This path is used for detection and localization. The U-Net was trained with the images of a dataset using a determined number of epochs. Multiple epochs are necessary because U-Net uses gradient descent for learning optimization (Kirk, 2014). To present the performance of the algorithm on our dataset, we use the Jaccard index IoU score. It indicates the overall accuracy and is calculated as the intersection of the segmented areas between the ground truth and the prediction over the union of both areas. Consequently, the ratio of incorrectly classified pixels per total classified pixels is depicted by the Jaccard loss function. To create a segmented image, the U-Net algorithm was trained with the obtained images and tested on an image different from the training dataset. The segmentation using the U-Net was carried out via Python using PyCharm Community Edition 2018. The spectral analysis was executed using MATLAB R2018a.

## Results

**FIGURE 2:**
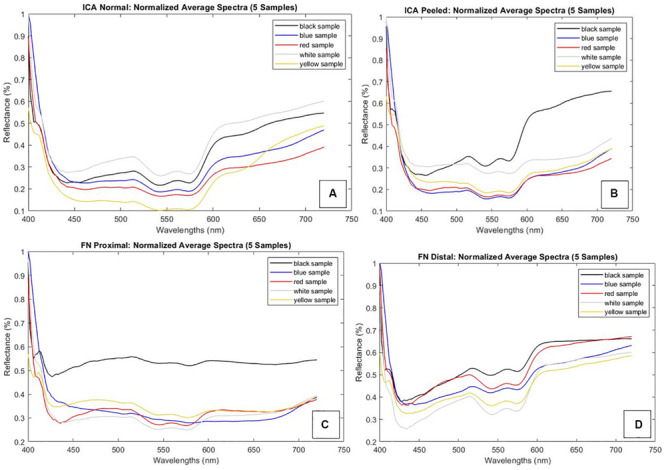
Normalized spectral signatures of studied tissue types and a mention of sample-specific anomalies. Graph corresponding colors (black, blue, red, white, and yellow) are used to identify the set of samples. Each set constitutes ICA peeled, ICA normal, FN distal, and FN proximal samples. **(A)** ICA normal samples. Tunica adventitia overlaps on yellow samples with lower reflectance. **(B)** ICA peeled samples. Plaque formation on black samples with notably higher reflectance values. **(C)** FN proximal samples. Unknown discoloration on black samples with a flat curve. **(D)** FN distal samples. Abbreviations: ICA, internal carotid artery; FN, facial nerve.

Each of the four types of tissue sample annotations and corresponding spectral signatures are provided in Figure 2. We observed a few distinct anomalies: overlapping of the ICA tunica adventitia in removal and notable atherosclerotic plaque formation were clearly visible in the spectra of ICA tissues. These abnormalities effected the reflectance curve either by distorting the shape (plaque) or altering reflectance levels (overlap). Proximal FN sample 1 appeared discolored during the extraction, which was likely due to unknown degenerative processes. The anomalies suggest identifiable changes in tissue-specific spectra; however, the phenomenon requires further studies.

The Mann–Whitney *U*-test was used for all statistical analyses as the assumption of normality was not fulfilled for every tissue. The ICA with intact tunica could be reliably differentiated from samples with peeled tunica on lower (400–474 nm) and middle (532–556 and 572–576 nm) wavelength bands (*p* < 0.05). Proximal FN could be differentiated from the distal part in the lower (408–416 nm) and medium to high (490–720 nm) wavelength bands (*p* < 0.05 and *p* < 0.001, respectively). The proximal of the FN was also compared to the normal ICA, indicating significant differences throughout 412–592 and 664–720 nm (*p* < 0.001 and *p* < 0.05, respectively). A similar comparison with the distal part of FN illustrated significant differences nearly throughout the screened wavelengths (422–720 nm, *p* < 0.001). The complete Mann–Whitney test results with respective test parameters are presented in Table 1. Similarly, the spectral pairwise comparisons indicated the strongest separatory capabilities on blue to green and red wavelength regions.

**TABLE 1:** Significant wavelength ranges and reflectance characteristics of the tissue groups.

	**ICA normal–ICA peeled**	**FN proximal–FN distal**	**FN proximal–ICA normal**	**FN distal–ICA normal**
Mann–Whitney test ranges	1: 400–474 nm 2: 532–556 nm 3: 572–576 nm	1: 408–416 nm 2: 490–720 nm	1: 412–592 nm 2: 664–720 nm	1: 422–720 nm
*p*-value	*p*_1_ < 0.05 *p*_2_ < 0.05 *p*_3_ < 0.05	*p*_1_ < 0.05 *p*_2_ < 0.001	*p*_1_ < 0.001 *p*_2_ < 0.0	*p*_1_ < 0.001
Medians	A 0.179	B 0.194	C 0.214	D 0.290
Percentiles and IQR	A *Q*_1_ = 0.130 *Q*_3_ = 0.250 IQR = 0.120	B *Q*_1_ = 0.153 *Q*_3_ = 0.235 IQR = 0.082	C *Q*_1_ = 0.184 *Q*_3_ = 0.271 IQR = 0.087	D *Q*_1_ = 0.235 *Q*_3_ = 0.348 IQR = 0.113
Normality of ranges	A 406–720 nm	B 406–500 nm	C 408–436 nm	D 408–720 nm

*(**A**) ICA normal, (**B**) ICA peeled, (**C**) FN proximal, and (**D**) FN distal. Abbreviations: ICA, internal carotid artery; FN, facial nerve; IQR, interquartile range.*

**FIGURE 3:**
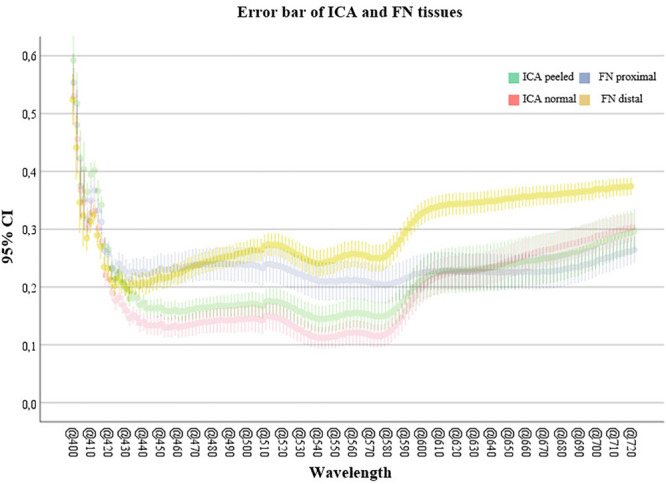
Confidence interval (95%) error bars comparing artery and nerve groups. The highest mean difference is illustrated in the lower and middle wavelength ranges (400–590 nm) between ICA samples and middle to high ranges (490–720 nm) for FN samples. Abbreviations: CI, confidence interval; ICA, internal carotid artery; FN, facial nerve.

Illustrations of the mean differences between ICA and FN groups are plotted with 95% confidence interval error bars in Figure 3, which also describes the significant wavelength regions that were confirmed statistically. In all, the error bars indicate minor data spread around the group means.

The U-Net algorithm was trained on 68 of the 161 bands in the image, in less than 20 min on a laptop with 16 GB of RAM. Generating the predicted classification took around 12 s on the same machine. The IoU score and Jaccard loss function indicated that overall accuracy increased rapidly up to 25 epochs but flattened above 80 epochs and that further increase did not improve segmentation significantly. At the end of the training, the accuracy reached 90.93% and the loss around 10%. Figure 1B shows the output of the carried segmentation in the context of imaged tissues.

## Discussion

We have assessed the spectral behavior of ICA and FN in the visible wavelength domain. It is indicated that the applied LCTF spectral system allows for classification of neurovascular structures and their characteristics in *ex vivo* conditions. Consequently, we can bring forth the spectra for the predefined tissues and find the significant wavelengths for differentiating between tissues. Similarly, we were able to differentiate within tissues’ microstructural changes related to anatomical location and iatrogenic conditions—which are associated to tissue manipulation during surgical approaches.

Both low (400–490 nm) and high (585–720 nm) regions of the VIS spectrum appear as the most significant intervals for *ex vivo* artery and nerve features, whereas the middle (490–585 nm) seems less informative from a computational perspective. As to the ICA-vs.-FN comparison, the whole VIS range demonstrated robust capability to differentiate between the two. However, wavelengths close to the SI system’s calibration limits (400 and 720 nm) may be prone to bias, so the low–middle (450–585) wavelengths seemed most feasible for applications focusing on ICA. When FN is investigated, the most feasible wavelengths were focused on longer VIS wavelengths, namely, above 644 nm. We did not compare these samples to any other tissues in this study; however, the medium-to-high spectral region has been found most relevant earlier in *ex vivo* temporal bone and soft tissue studies (Bartczak et al., 2018; Wisotzky et al., 2018).

In earlier studies, connective tissue has revealed great variability; this may result from different connective tissue types or biological processes (Wisotzky et al., 2018). In our study, the optic spectra of the well-defined neuroanatomical tissue samples demonstrated modest normal variance at most. Reflectance values of ICA normal samples demonstrated a median of 0.179 [interquartile range (IQR) = 0.250–0.130], ICA peeled 0.194 (0.235–0.153), FN proximal 0.214 (0.271–0.184), and FN distal 0.290 (0.348–0.235). Most importantly, excluding the samples with anomalies, the graphs presented a uniform shape between individuals. These findings suggest that ICA and FN tissues’ VIS range features could be individually extrapolated from narrow-band illuminations. As the SI system calibration is a critical and time-consuming step for optimizing visual output, this individual consistency is a noteworthy phenomenon for improving performance on personalized optic visualization systems. Larger sample size studies could be used for creation of multispectral sensor fabrication where number and type of optical properties of the filter would correspond to some of the significant bands. This could result in much better segmentation or recognition when comparing with conventional methods (RGB). As another advantage, reduced number of filters could deliver real-time imaging and segmentation.

Wisotzky et al. developed a band-pass filter wheel-based hyperspectral camera setup to monitor the different optical behaviors of tissue types *in vivo* under white light. They evaluated the artery, vein, bone, muscle, fat, connective tissue, parotid gland, and nerve from six (*n* = 6) different patients, and HS data were acquired in three different surgical procedures: mastoidectomy, parotidectomy, and neck dissection (Wisotzky et al., 2018). The acquired results showed that the behavior of the normalized reflection intensity for the analyzed tissue type remains the same for different measurements and individuals in the analyzed spectrum from 400 to 700 nm. Consistent to our findings, their results showed individual trends for each tissue type, which allows precise identification during the operation.

Preserving ICA and FN along with their anatomical passageways are important sites for temporal and neurovascular microsurgery. As presented in Table 1, peeling of ICA objectively alters the reflective properties of the arterial walls with most significant *ex vivo* differences at low band regions. Identification of arterial wall breaches with optimized illumination could, for example, improve the ability to predict imminent complications such as vasospasm, which indicates treatment with antispasmodic agents such as papaverine. On an *in vivo* setting, blood perfusion will likely dominate arterial spectral features; however, our observations on low band (blue) differences seem nevertheless promising as the reflective properties of blood concentrate on the higher bands (red) and up to near-infrared (NIR) levels (Bosschaart et al., 2014). In our study, we also observed anomalies, namely, the effect of atherosclerotic plaque on the artery spectra, which had a clearly different signature. Hence, the amount and type of surrounding tissue results in varying reflectance values and requires further analysis. With a larger sample size, histopathological analysis would be a relevant method to investigate the outliers such as the ICA samples affected by macroscopic atherosclerotic plaque. Optimized illumination of FN can assist its early detection in subtemporal and intraosseal surgery (Coker et al., 1987) and help avoid iatrogenic damage. For middle and posterior fossa skull base surgery, the early detection of intracranial nerves such as FN offers similar support for patient safety, especially on complicated conditions involving adhering lesions such as tumor infiltrations, blood, and gliosis. We observed that the optic spectrum of FN is moderated by its microanatomical location, and although lacking histological confirmation, we hypothesize that the difference is due to peripheral nerve myelination in comparison to its intracranial counterparts.

Degenerative processes occur in human tissue both with and without blood supply, in addition to alterations in water and collagen concentrations. Hence, the optical properties are expected to change especially between cadaveric *ex vivo* and *in vivo* blood-perfused samples. This change in optic features should be considered a limitation when translating our *ex vivo* results to live tissue.

The potential clinical advantages of different SI and NBI solutions provide a window for thoroughly describing healthy and pathologic tissues’ optic signatures. As demonstrated previously, combining SI and classification algorithms has successfully enhanced segmentation of tissues in different surgical trials. Recently, Fabelo et al. (2018b) captured spectral images from the brain surface and conducted an automated analysis that resulted in accurate identification of glioma borders (*n* = 5) compared by margins delineated by specialists. The authors also propose collection and documentation of spectral database for research and imaging system development purposes. As presented here, computationally credible automatic segmentation of microneuroanatomical tissues was established, suggesting potential applications also in the microsurgical environment. The presented image segmentation by using the U-Net algorithm yielded over 90% overall accuracy for the obtained images. These findings provide perspective to applying SI technology to tissue-specific microsurgical research as automated tissue segmentation is still in its infancy. In our study, tuned spectral image cubes were used to train U-Net to differentiate between microsurgical tissue samples. After the system was trained, the performance of the U-Net visual segmentation was accurate and appeared fast enough for intraoperative settings. The determined most significant bands were used together with the U-Net and point out the importance of optimizing the number and type of the spectral bands for high-accuracy automatic segmentation. During near-real-time application, the immersive spectral data will benefit from calibration assistance with automated analyses such as U-Net, which turned out to be reliable and fast in our setting. However, the acceptable threshold for error should be determined by each utilizing expert case-specifically.

## Conclusion

This study indicates that SI systems are feasible for augmenting identification of delicate neuroanatomical tissues and their substructures. Our NBI screening protocol introduces insight for further development of advanced optic imaging systems focusing on identification of characteristic structures such as intracranial arteries and cranial nerves. Such systems may help conserve normal anatomy by assisting in their early detection and potentially help identify and counteract complications due to microstructural trauma caused by tissue manipulation during skull base surgeries. We observed that optical properties of medical samples link to the type of tissues’ anatomical locations and condition. These observations set a requirement for an extensive anatomical spectral library of healthy tissues. Future considerations include studies of different intracranial tissues, larger sample sizes, and samples with circulation. In addition, hyperspectral bands such as ultraviolet and NIR wavelengths need to be included to the optic screening protocols.

Evidently, one of the principal limitations in the development of intraoperative SI systems is the lack of overt, tissue-specific spectral data. The creation of an internationally available spectral database of different surgical pathologies is an international and multidisciplinary objective, as previously suggested by Fabelo et al. Future studies at the Eastern Finland Microsurgery Center are working toward this long-term goal. Intraoperative spectral video application that fulfills ergonomic and sterility requirements of operation rooms is also under future development in the center. Decidedly, the expertise in computational field is vital for the blossoming of intraoperative SI. In the future, multidisciplinary research is essential for successful integration to the operating room workflow.

## Data Availability Statement

The datasets generated for this study are available on request to the corresponding author.

## Ethics Statement

The studies involving human participants were reviewed and approved by the Ethics Committee, Hospital District of Northern Savo. Written informed consent for participation was not required for this study in accordance with the national legislation and the institutional requirements.

## Author Contributions

SP made substantial contributions to the conception and design of the work, the image acquisition, analysis, interpretation of data for the work, and writing the complete research report. SA made substantial contributions to the analysis and interpretation of the data and drafted the “Computational Methods” section. RB and SA made substantial contributions to the conception and design of the work. PB made substantial contributions to the image acquisition and data analysis and revised the work critically for important intellectual content. RB, TK, AD, and MF revised the work critically for important intellectual content. MI-M revised the work critically for important intellectual content and preparation of imaged samples. A-PE made substantial contributions to the conception and design of the work and revised the work critically for important intellectual content. All authors agreed to be accountable for all aspects of the work in ensuring that questions related to the accuracy or integrity of any part of the work are appropriately investigated and resolved, and provided approval for publication of the content.

## Conflict of Interest

The authors declare that the research was conducted in the absence of any commercial or financial relationships that could be construed as a potential conflict of interest.
